# Risk Factors of Intervertebral Disc Pathology—A Point of View Formerly and Today—A Review

**DOI:** 10.3390/jcm10030409

**Published:** 2021-01-21

**Authors:** Nicol Zielinska, Michał Podgórski, Robert Haładaj, Michał Polguj, Łukasz Olewnik

**Affiliations:** 1 Department of Anatomical Dissection and Donation, Medical University of Lodz, 90-752 Lodz, Poland; nicol.zielinska@stud.umed.lodz.pl; 2 Department of Radiology and Diagnostic Imaging, Polish Mother’s Memorial Hospital Research Institute, 93-338 Lodz, Poland; chilam@o2.pl; 3 Department of Normal and Clinical Anatomy, Chair of Anatomy and Histology, Medical University of Lodz, 90-752 Lodz, Poland; robert.haladaj@umed.lodz.pl (R.H.); michal.polguj@umed.lodz.pl (M.P.)

**Keywords:** review, disc herniation, lumbar, cervical, thoracic, factors, prevention, statistics

## Abstract

Intervertebral disc pathology is a common disorder that can be caused by genetic, mechanical, and behavioral factors; however, it is possible to slow its progression. Although environmental and behavioral factors were previously considered to be the sole causes of intervertebral disc pathologies such as disc herniation, recent studies have shown that genetic factors also play an important role. This review compares the perception of major risk factors from the last and present centuries. It also examines individual genetic and non-genetic factors acting as risk factors, as well as some approaches for preventing intervertebral disc pathologies, and compares available statistics regarding disc herniation.

## 1. Introduction

The spinal column consists of a series of intervertebral discs and vertebrae that originates at the base of the skull and extends to the coccyx. The complete structure is typically divided into five parts: the cervical region (C1-C7), the thoracic region (T1-T12), the lumbar region (L1-L5), the sacral region (S1-S5), and the coccygeal region (Co1-Co4/5). It provides flexibility and mobility for the body and gives protection to the spinal cord [1].

**Figure 1:**
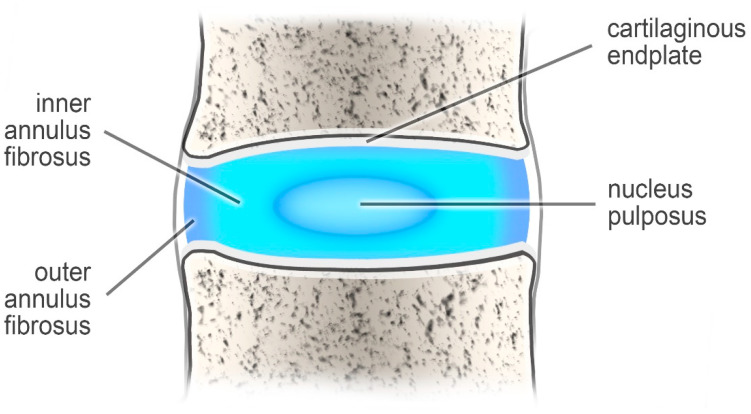
The intervertebral disc.

The intervertebral discs support the spinal column and behave like shock-absorbing cushions against the axial loading of the human body [1]. These structures are built of two components: a nucleus pulposus (which is the inner part) and an outer annulus fibrosus (AF) [2] (Figure 1).

The nucleus pulposus, which consists of collagen (type II), makes up 20 percent of the dry weight of the disc. It also contains numerous proteoglycans that maintain hydrostatic pressure by retaining water. The other ingredients are water and chondrocyte-like cells. This composition helps the nucleus pulposus remain elastic, capable of absorbing compression, and flexible under stress forces [2,3].

In turn, the annulus fibrosus is formed from collagen type I, which forms a fibrous tissue around the nucleus pulposus. The annulus fibrosus is attached to the vertebral body by Sharpey fiber [3].

Intervertebral disc pathology is a common name for various pathologies connected with intervertebral discs. This group includes degeneration of discs, disc-associated pain, disc herniation, and histology findings. Disc degeneration can be graded on MRI T2 spin-echo weighted images using a grading system (grade 1–5) proposed by Pfirmann [4]. Grade 1 is represented by an inhomogeneous disc with bright hyperintense white signal intensity and normal disc height. In turn, grade 5 disc degeneration is characterized by an inhomogeneous disc with a hypointense black signal intensity. There is no clear distinction between the AF and NP, and the disc space is collapsed [5]. There are also histological findings associated with disc degeneration. An increase in cell density (chondrocyte proliferation), the occurrence of granular changes, structural alterations with tears and clefts, and a severe increase in acid mucopolysaccharides (mucous degeneration) with dark-blue staining areas around clones of chondrocytes are the main characteristic features observed during histomorphological examination [6]. Another pathology that can be included in intervertebral disc pathologies is low back pain. However, it may be caused not only by disc herniation, but also by infection, rheumatoid arthritis, fracture, osteoporosis, or tumors [7].

Disc herniation is an intervertebral disc pathology that occurs when either part of the whole of the nucleus pulposus protrudes through the annulus fibrosus. The most common cause is chronic herniation, i.e., disc degeneration occurring due to reduced proteoglycan content, in which the nucleus pulposus is weakened due to a loss of hydration. In contrast, acute herniation can occur as a result of trauma, resulting in the nucleus pulposus extruding through a defect in the annulus fibrosus. Many genetic, mechanical, and behavioral factors are believed to be responsible [8]. MRI is a first-choice examination method. It allows disc herniations to be classified according to their shape or extent and distinguishes three types: protrusion (largest diameter of the disc material projecting beyond the normal margin of this disc is narrower than the general width of the base), extrusion (characterized by a base length less than the height), and sequestration (a subtype of extrusion, which occurs when there is no connection between the herniated material and the intervertebral disc) [9]. There is also a disc bulge (which involves more than 25% of the circumference), another kind of discopathy that MRI also reveals. It is important that the prevalence of disc bulge in asymptomatic individuals increases from 30% of those 20 years of age to 84% of those 80 years of age. In turn, more advanced disc herniations usually compress neural structure, which causes various types of pain [10].

Due to the biomechanical forces in the flexible parts of the spinal column, the cervical and lumbar regions of spinal column are more susceptible to occurrence of disc herniation. Thoracic disc herniation is much less common [8].

## 2. Embryology of Vertebrate

Ossification occurs in all vertebrae after around eight weeks of embryo development. It occurs in three centers: one in the endochondral centrum and two in neural processes. The process in endochondral centrum is responsible for the development of vertebral bodies, while those in the neural processes are associated with formation of pedicles. Ossification begins in the thoracolumbar part of the spinal column and proceeds in the cranial and caudal directions [11].

Between the ages of three and six, neural processes fuse with the centrum. This is followed by the appearance of five secondary ossification centers: one at the tip of the spinous process, two on the transverse processes, and one on both the superior and inferior surfaces of the vertebral bodies [11].

The superior-inferior growth of the vertebra occurs due to presence of ossification centers localized on the vertebral body. The ossification process ends at about the age of 25 [11].

During embryological development, the nucleus pulposus is formed by the notochord. It is responsible for conserving energy during compression. It also acts with other disc substructures (the annulus fibrosis and cartilaginous end plates) during spinal movements [12]. It is worth mentioning that there are some genes involved in vertebral column development. HOX genes encoding homeodomain proteins (transcription factors) take part in anterior-posterior body patterning and vertebra number [13]. The development of the spinal structures is regulated by numerous factors that interact with each other. For example, Shh (Sonic Hedgehog) changes cellular responses to bone morphogenetic proteins. A chondrogenesis of the nascent disc is the effect of this process. TGF-β and Nog also take part in this formation [14]. Genes involved in vertebral column development may also contribute to its maintenance, degeneration, and regeneration in adults. Polymorphism or mutations of these genes may correlate with disc degeneration and back pain [15].

## 3. Factors Formerly and Today

Disk herniation is known to be caused by genetic, mechanical, and behavioral factors. However, even recently, this was not believed to be the case, with previous models of dysfunction being based on environmental and behavioral factors [16]. These were later disproven [17].

It was believed that the main causes of disc herniation were aging and mechanical insults, and that smoking status, aging, obesity, height, sciatic axial overloading, or occupation had the greatest influence on the presence of intervertebral disc pathology [17]. Although both obesity and height can be classified as anthropometric measurements, the latter is rarely raised in studies [18]. Even in 1999, the prevailing belief in many countries was that work-related mechanical factors like heavy physical loading, lifting, bending, and twisting played the main role in lumbar degeneration [19].

Environmental, i.e., mechanical and behavioral, factors associated with occupation (physical workload, hard work, a working period longer than eight hours, and stress at work) are believed to be the major factors in intervertebral disc pathology [20].

A number of studies from the last century focused solely on the environmental factors of intervertebral disc pathology, with no mention of the possible influence of genetic factors. For example, one study found that men who drove a motor vehicle for half or more of their working time were three times more vulnerable to acute lumbar herniation than other people; however, this study did not assume any possible genetic predisposition [21]. Another study proposed that the main mechanism in the degeneration of the intervertebral disc was the nutritional decline of the central disc. This was suggested to lead to collection of waste products, the degeneration of matrix molecules, and a fall in pH, which may cause cell death [22]. This is true, but later studies demonstrated that polymorphisms or mutations among genes associated with substances of connective tissue also accelerated the degeneration of intervertebral disc.

A 1992 study included the contribution of genetic factors; however, these were believed to play a lesser role than environmental factors such as age, gender, cigarette smoking, exposure to vehicular travel, and occupation [23].

However, the use of new genetic and proteomic tools has allowed the effects of genetic variation to be researched. They made it possible to link genetic variations with the occurrence of different diseases and molecular-level processes to pathologies such as disc herniation [17]. These new molecular studies cast doubt on the previous belief that environmental factors are the main (or only) cause of intervertebral disc pathologies. Later, in 1998, a number of gene alterations were found to be associated with disc degeneration [24].

Over the years, the classical theory of aging and wear-and-tear evolved into a sophisticated model of a multiple-causative disease based on molecular and genetic alterations [17,25]. Polymorphisms or gene mutations were found to be associated with disc herniations in both humans and animals [17,25].

A number of new studies have confirmed that genetic anomalies are critical contributors to the initiation and further development of intervertebral disc pathologies [17,24,26,27,28,29].

The Twin Spine Study, a multinational research project that followed twins from Canada, Finland, and the United States, examined how occupational exposure, vibrational exposure, smoking, anthropomorphic characteristics, and genetics affect the occurrence of disc degeneration. The findings indicated that pairs of identical twins with different jobs, physical activity, and interests demonstrated similar levels of disc degeneration, suggesting that genetic factors have much larger influence on disc degeneration than expected [16].

Another study of 75 monozygotic twin pairs that were examined twice, with five years in between measurements, found familial aggregation, i.e., genetic and environmental factors that were identical between twins, to explain 47% to 66% of the variance in progression of degenerative signs on lumbar MRI. In turn, environmental factors such as occupational physical loading and resistance training explained only 2% to 10% of the progression in the degenerative signs in lumbar MRIs. The main conclusion of this research was therefore that factors associated with genes play a dominant role in disc degeneration, while environmental factors only have a modest one [30].

However, a 2009 study by Battié et al. of 152 monozygotic and 148 dizygotic male twin pairs [31] indicated that genetic factors only have a partial influence on the development of disc degeneration, according to phenotype and spinal level. Estimates of heredity varied from 29% to 54%, depending on the phenotype and spinal level. In turn, disc narrowing and bulging had a mainly common genetic pathway [31].

In addition, Videman and Battie reported that the influence of occupational risk factors and occupation on the occurrence some intervertebral disc diseases was also modest, especially in comparison with contribution of genetic factors [19]. Kepler et al. [29] reported that genetic factors account for up to 75% of individual susceptibility to intervertebral disc degeneration, and that environmental factors such as occupational exposure and smoking do not have the greatest impact on disc pathologies. In addition, a study of the effects of anthropometrics, lifting strength, and physical activities on disc degeneration found that occupational and leisure physical activity, body weight, and lifting strength have a low impact on occurrence of disc degeneration [32].

A study of 63 patients under the age of 21 with confirmed lumbar disc herniation found 32% to have a positive family history. In contrast, the prevalence among the control group was only 7%, i.e., approximately one fifth of the group with a positive family history [33]. Hence, genetic factors may play a significant role.

A recent systematic review about genetic predisposition to disc degeneration by Filho et al. found that genes play the main role in the occurrence of disc degeneration, and that environmental factors have less importance. Obesity, occupation, smoking status, diabetes, and alcohol consumption were described as aggravating factors. Hence, disc degeneration appears to be a multifactorial process, but with genetic factors having the greatest influence [34].

A case-control study of risk factors for herniated disc in the lumbar region, including BMI, smoking index, level of education, occupation, socio-economic status, intensity of physical labor at home and work, and self-assessed limitation in physical activity, was performed on a population from Croatian Islands. Hereditary factors were found to be strongly associated with disc herniation; hence, such isolated populations appear to be valuable for detecting such effects due to decreased genetic and environmental variability. The findings indicated that a number of environmental factors, such as level of education, cardiovascular morbidity, smoking status, intensity of physical work, or socio-economic status, did not have a meaningful influence on the occurrence the lumbar intervertebral disc herniation in this population [35].

Genetic factors appear to play the main role in pathology of diseases associated with disc degeneration, and they may be influenced by environmental factors such as spine injury, occupation, smoking, or aging [29] In turn, environmental factors may only serve to accelerate this process of disc degeneration driven by genetic polymorphisms.

Although these findings provide a detailed picture of the etiology of intervertebral disc degeneration, there are still many unsolved issues connected with the magnitude and mechanism of genetic factors. In addition, the studies had various limitations that may have affected the correctness of some research results. For example, some studies used a small cohort, resulting in possible false-positive or false-negative errors.

In addition, to design genetic therapy, future studies should carefully examine the loci of genes connected with intervertebral disc degeneration. Such progress would be supported by progress in molecular biology and advancements in imaging technology, allowing faster diagnosis of intervertebral disc degeneration [17].

Regarding individual risk factors influencing disc degeneration, both environmental and genetic factors interact in the formation of disc herniation. Although intervertebral disc disease has multiple causes, the key role appears to be played by changes in genes at the molecular level [16,17].

## 4. Genetic Factors

Many genes are believed to take part in processes associated with disc herniation. They are responsible for encoding matrix metalloproteinases, structural proteins, and growth and apoptosis factors. Additionally, single nucleotide polymorphisms in the vitamin D receptor gene, resulting in inflammatory cytokine imbalance, also increase probability of disc herniation [2].

### 4.1. Vitamin D Receptor

Vitamin D plays a key role in the sulphation of glycosaminoglycans, which is connected with proteoglycan synthesis; as such, polymorphisms in the vitamin D receptor (VDR) gene may have an effect on the level and function of this receptor. Such changes can influence the structural characteristics of the extracellular matrix in the intervertebral disc [36].

The vitamin D receptor (VDR) belongs to the group of steroid nuclear receptors. The study of the VDR polymorphisms—*Taq*I (located in a noncoding region of Exon 9) showed that this polymorphism is connected with disc herniations. A study of 209 Japanese people (age: 20–29) confirmed that disc herniation occurs more frequently in groups of people with *Tt* genotype of the *Taq*I polymorphism than the group with the *TT* genotype [17,37]. In addition, Videman et al. [38] assessed the intensity of MRI signals and found that the intensity in thoracic and lumbar discs in men with *Taq*I tt and Tt genotype were worse by 12.9% and 4.5%, respectively, than men with the *TT* genotype [38].

The FokI polymorphism, located in the region of Exon 2, eliminates the first translation initiation codon (ATG) and allows the second start codon to be translated. Patients with the ff genotype demonstrated a 9.3% weaker MRI signal, and those with Ff 4.3% weaker, compared to patients with FF genotype [17,37,39].

### 4.2. Collagen Type I

Type I collagen is encoded by two types of genes, *COL1A1* and *COL1A2*, which are present in both the nucleus pulposus and annulus fibrosis. A mutation in *COL1A1* was found to increase the risk of disc herniation in a study of 966 patients (age > 64). People with the *TT* genotype were more prone to intervertebral disc degeneration than those with GG and GT genotypes. Hence, the presence of a polymorphism in *COL1A1* may lead to spinal pathology [40].

Similar results were observed in another study of Greek military recruits. No healthy participants had a *TT* genotype for *COL1A1*; however, this genotype was observed in 33.3% participants with lumbar disc disease. Hence, the presence of *COL1A1* polymorphism may be associated with a greater chance of disc degeneration related to disc herniation [17].

### 4.3. Collagen Type IX

*COL9A1*, *COL9A2,* and *COL9A3* encode collagen type IX, which is a heterotrimeric protein consisting of three genetically distinct chains (α1, α2, and α3). Several distinct studies indicate that mutations in these genes are associated with disc degeneration [17,37].

The first example is a study conducted on mice. The results showed that inactivated *COL9A1* and overexpression of mutant *COL9A1* affected accelerated disk degeneration and more herniation. The research sample was compared to the control sample with correct expression of *COL9A1* [17].

The Trp2 allele is a rare *COL9A2* allele that replaces the arginine in the protein with tryptophan. Such mutations lead to changes in disc structure such as annular tears and end-plate herniations, with odds ratios of 2.4 and 4.0, respectively. The inheritance of Trp2 was found to be associated with the presence of intervertebral disc disease in the family of all patients [17].

Arg103Trp is an amino-acid substitution of the Trp3 in the third chain of collagen IX. This mutation increases the risk of intervertebral disc disease. A Finnish study of 164 patients found 40 to have the Trp3 allele (24%), compared to only 9% of healthy controls [41]. Another study also showed the relationship between the presence of Trp3 allele and occurrence of intervertebral disc disease. Arg103Trp substitution was found to be present in 12.3% of subjects with disc disease, but only 4.7% of healthy controls [42].

### 4.4. Collagen Type XI

Type XI collagen is a cartilage-specific ECM protein used in the organization of the ECM and cartilage-collagen fibril formation. Its three chains, encoded by *COL11A1*, *COL11A2*, and *COL11A*3, are involved in procollagen synthesis, which regulates the diameter of cartilage collagen fibrils. A single-nucleotide polymorphism in *COL11A1* has been found to have a significant relationship with lumbar disc herniation; in addition, decreased expression of *COL11A1* has been associated with lumbar disc herniation and a greater severity of degeneration [43]. Mutations in Intron 9 of *COL11A2* may also lead to an increased risk of disc bulges [44].

### 4.5. Aggrecan

Aggrecan is a proteoglycan ingredient that contains a large number of chondroitin sulfate chains and builds the nucleus pulposus. Its main function is to provide compressive strength. Aggrecan is encoded by *ACAN* (also known as *AGC1* or *AGCAN*). The gene has a variable number tandem repeat (VNTR) section in Exon 12 (size polymorphism). Changes in the lengths of the VNTR may result in differences in aggrecan properties. A study of 64 young women with or without low-back problems found that a shorter VNTR length may predispose multilevel disc degeneration [45].

### 4.6. Matrix Metalloproteinase-3 (MMP-3)

Matrix metalloproteinase-3 is an enzyme responsible for the degradation of the disc matrix. It takes part in devastation of proteoglycans and collagen fibers. Its expression can be induced by inflammation and mechanical factors. The 5A/6A polymorphism of the human matrix metalloproteinase 3 gene has been found to have an influence on MMP-3 regulation. Takahashi et al. [46] found the presence of the 5A/6A polymorphism to be related to an acceleration of intravertebral disc degeneration in elderly subjects, which may lead to disc herniation. The presence of 5A/5A and 5A/6A genotype resulted in a much higher frequency of disc pathology than in the case of 6A/6A, suggesting that the 5A allele is a possible risk factor [46].

### 4.7. Interleukin-1

Interleukin-1 is a cytokine produced during inflammation, injury, and antigenic challenge. The activity of these molecules can affect the metabolic and neurologic system. The Il-1 gene family contains IL-1α, IL-1β (strong inducers of inflammation), and IL-1RN (suppressor of IL-1-competition inhibitor of binding IL-1 to its receptors). An increased level of interleukin -1 was noticed in the presence of the IL-1β + 3954 T allele or the *TT* genotype (instead of the CC genotype) of the IL-1α (-889C→T) promoter polymorphism [47]. Il-1 takes part in the expression of matrix metalloproteinases, for example MMP-3, which is responsible for the degradation of the ECM (proteoglycan-like aggrecan in disc). Due to this, the efficiency of collagen and proteoglycan synthesis is decreased [48]. A study that investigated the association between intervertebral disc degeneration and polymorphisms in genes encoding IL-1 showed that the odds ratio for disc bulges was 2.4 and 3.0, in carriers of the IL-1β + 3954 T and IL-1α -889 T a, respectively [44]. Another interesting hypothesis indicated the relationship between functional SNP (+3954C→T) in Exon 5 of the IL-1β gene and pathologies in disc structure, because this polymorphism can interreact with other gene risk factors such as the Trp3 allele [44], which is also associated with environmental factors such as obesity [37].

### 4.8. Interleukin-6

Interleukin-6 is a proinflammatory cytokine that plays a role in discogenic pain. The presence of an “A” allele in an IL-6 SNP in Exon 5 increases the risk of pathology. The association of GGGA with disease was highly significant and confirmed by a study conducted by Noponen-Hietala et al. [49,50]. Another study regarding the genetic risk factors of disc degeneration among 12–14-year-old Danish children also showed that polymorphisms in genes that encode interleukin-6 are connected with disc degeneration, which may lead to disc herniation. The C-allele (in IL6 promoter polymorphism rs1800796) was more frequent among the subjects with disc degeneration observed during MRI [51].

### 4.9. Cartilage Intermediate Layer Protein

Cartilage intermediate layer protein (CILP) is a matrix constituent of human articular cartilage. CILP plays a role in lumbar disc degeneration by regulating the TGF-beta signaling pathway. CILP inhibits the TGF-β1-mediated induction of extracellular matrix proteins (for example, aggrecan and collagen II), through direct interaction with TGF-β1. Cartilage intermediate layer protein is expressed in intervertebral discs. Its expression has an impact on disc pathologies. A study that confirmed these statements was carried out by Seki et al. [52], which included a total of 467 Japanese men and women with disc degeneration in the research sample. In turn, the control sample consisted of 654 people. In the research sample, every patient suffered from unilateral back pain radiating to a leg. They underwent MRI examination. In total, 367 cases of this population required surgery for lumbar disc herniation [52,53].

### 4.10. Other Genes

There are also other genes that are associated with intervertebral disc pathologies like disc herniation. MMP-2 (matrix metalloproteinase-2) takes part in the pathophysiology of lumbar disc disease. The polymorphism -1306C/T in the promoter region affects the increased frequency of transcription, and leads to higher expression that results in degenerative discs [54]. Another matrix metalloproteinase (MMP9) was tested by Sun et al., and the occurrence of a CT/TT genotype resulted in a twofold increased risk of intravertebral disc degeneration compared to individuals with a CC genotype [55]. We can also include TIMP1, COX2, and THSD2 genes in this group. Their polymorphisms are connected with intervertebral disc degeneration; which was confirmed by a study by the Chingford cohort [56]. Single-nucleotide polymorphism (SNP) in the insulin-like growth factor-1 receptor was investigated by Urano et al. [57] The study population was 434 Japanese postmenopausal women. A higher disc-narrowing score on radiographs was associated with the presence of the G allele (GG or GC genotype) in the insulin-like growth factor-1 receptor; genetic variation at the IGF1R gene locus caused disc degeneration [57].

DNA methylation may be also involved in pathogenesis of intervertebral disc degeneration. DNA methylation is a process in which methyl groups are added to a cytosine in a CpG-containing nucleotide to form 5-methylcytosine. It causes changes in gene expression, but not in the sequence of the DNA [5]. Another study [58] compared the early and advanced stages of degenerated discs. The results showed that 220 loci were differentially methylated. It is a total of 187 individual genes. Moreover, the early and advanced stages of degeneration of human nucleus pulposus tissues exhibited substantially various methylomes. The vast majority of loci in the advanced stage were hypermethyled (216 loci). Only four loci were hypomethylated. For example, in the differentially methylated loci, the averaged β value of SNORA52, GNL3, and MED23 was significantly higher in the advanced stage compared to the early stage. Moreover, there was an association between the occurrence of disc degeneration and differentially methylated genes that are connected with signaling pathways (NF-κB, MAPK-ERK, and Wnt signaling pathways). In many genes associated with disc degeneration, differentially methylated loci were noticed, confirming that DNA methylation takes part in intervertebral disc pathologies [58].

## 5. Factors Not Connected with Genes

There are also risk factors that are not connected with gene polymorphisms. This group includes smoking status and aging. There are also factors connected with anthropometric measurements: obesity (which is closely related to BMI) and height (the factor of which is rarely raised in scientific research). Disc herniation also may be associated with occupation. We can distinguish four criteria connected with this factor: physical workload, hard work, a working period >8 h, and stress at work. Disc herniation also can be caused by static axial overloading, which can be observed among groups of people with a sedentary lifestyle. In this case, usually posterior disc herniation occurs. Environmental factors interact with genetic factors and accelerate the process of degeneration. Currently, there is no research in which the influence of environmental factors is be recognized as independent (without the influence of genetic factors).

### 5.1. Smoking

Smoking is a risk factor of disc herniation. This was confirmed by Andersen et al. [59] The research sample consisted of patients with lumbar disc herniation. The control group consisted of people without this pathology. Binary logistic regression analysis determined that smoking is an independent risk factor, accelerates disc degeneration, and promotes the development of lumbar disc herniation. Smoking causes the contraction of capillary vessels and leads to decreased diffusion of nutrition into the disc. Nicotine is a substance that works as an inhibitor of extracellular matrix synthesis and cell proliferation in the nucleus pulposus. Another study carried out by Akmal et al. [60] showed that collagen in the annulus fibrosis can be inhibited by nicotine. A decreased amount of collagen can be a predisposing factor to traumatic injury and degenerative changes leading to disc herniation [60] Holm et al. [61] indicated that smoking not only affects disturbed diffusion, but also significantly reduces the production of metabolites and deteriorates the cellular uptake rate within the disc [61].

### 5.2. Work

Heavy physical workload and occupations requiring harder effort are associated with increased occurrence of lumbar disc herniation. Ahsan et al. [20] performed a study in which 400 participants took part (200 in the research sample and 200 in the control sample). The results showed that the physical workload had the most significant influence. Other factors that affected the appearance of disc herniation were hard work, a working period >8 h, and stress at work [20]. Another study (comprising 267 cases with acute lumbar disc herniation and 197 control subjects) confirmed this statement; the study showed that extreme forward bending, cumulative exposure to the lifting of weights, and carrying are associated with lumbar disc herniation [62]. Zhang et al. [63] and Sun et al. [64] also noted the relationship between hard work and disc pathologies [63,64].

### 5.3. Static Axial Overloading

A disc herniation is not always associated with disc degeneration. There are patients who have a herniated disc caused by static axial overloading. Their sedentary lifestyle may lead to a specific subtype of herniations. This usually applies to younger people [2,65].

## 6. Connected or Not Connected with Genes?

There are also risk factors that are known to be determined by both genetic and environmental factors. These include aging, obesity, and height.

### 6.1. Aging

The process of disc aging is affected by many risk factors, such as genetic inheritance, obesity, nutrition, excessive mechanical loading, trauma, smoking, and inflammation, as well as catabolic cytokines and proteases. For that reason, we put aging in two groups: connected or not connected with genes. Compelling evidence has shown that the occurrence of disc herniation increases with age. Senescent cells accumulate in the disc, resulting in aging and degeneration. These cells inhibit proliferation, but they are still metabolically active. The blood vessels are located in the outmost layers of the annulus fibrosis. The rest of the annulus and the nucleus pulposus do not have blood vessels; hence, the cells are exposed to limited oxygen and nutrition supplies, leading to anaerobic metabolism connected with increased acidity [66].

### 6.2. Height

Height is a risk factor that is known to be determined by both genetic and environmental factors. A meta-analysis [67] identified 697 genome-wide significant SNPs in 423 loci that together explained one-fifth of the heritability for adult height [67].

However, a multitude of environmental factors also can affect height. Infancy is the most sensitive phase regarding external influences [68]. Adverse environmental conditions can decline the physical growth of children. The most important environmental factor is nutrition (for example, a lack of dietary protein). This group of environmental factors also includes childhood diseases. It shows that height is not only connected with genes, but also with behavioral factors [68].

Height has been found to influence the occurrence of disc herniation [69]. A group of 332 patients with lumbar disc herniation underwent anthropometric measurements. The control group contained 1205 individuals. Men with a height of 180 cm (the relative risk = 2.3) were compared with those whose height was more than 10 cm shorter. Women with a height of 170 cm or more showed a relative risk of 3.7 compared with a group who were more than 10 cm shorter [69].

### 6.3. Obesity

Obesity may be connected with genetic and behavioral factors. Andreasen et al. [70] carried out a study that showed that the obesity risk SNP in the FTO region was associated with obesity, but only among participants reporting little to no physical activity. Moreover, another study [71] showed that poor diet, deviation from a normal sleep pattern, and a socioeconomic deprivation score exaggerated the impact of the FTO region on BMI. For that reasons, we cannot accurately define whether obesity is a risk factor connected or not connected with genes.

Obesity (particularly the distribution of adiposity in the trunk of the body) is connected with biomechanical changes that cause a range of spinal diseases like disc degeneration, hypertrophy of the spinal ligaments, osteoarthritis, disc herniation, and spinal stenosis [72,73]. Rodriguez-Martinez et al. [74] confirmed this. Qualitative analysis using MR imaging showed the incidence of disc herniations occurred with increased frequency in an obese group versus controls [74]. A relationship has been found between BMI and the occurrence of disc herniation, but only among men [69].

## 7. Statistics

One of the main intervertebral disc pathologies is disc herniation. Some studies suggest that the frequency of herniated discs is influenced by non-genetic factors (for example: age group, gender, occupation).

A study carried out by Cheng et al. [75] investigated whether healthcare work is associated with increased occurrence of cervical disc herniation.

This result shows that physicians are more exposed to occurrence of cervical disc herniation than the reference group of non-healthcare providers. The same situation was observed in the case of non-physician HCPs and non-HCPs references, so we concluded that healthcare work is associated with an increased occurrence of cervical disc herniation (Table 1).

Table 2 shows whether physicians are more vulnerable to this pathology than non-physician HCPs. There was also a breakdown by gender.

We observed that generally, physicians are more vulnerable to the occurrence of cervical disc herniation than non-physician HCPs. Unfortunately, these dependencies were not statistically significant. The same situation occurred in the stratification analysis of gender.

Cheng et al. [75] also analyzed the occurrence of intervertebral cervical disc herniations among physicians according to specialization. The results are shown in Table 3.

We concluded that orthopedists are most vulnerable to the occurrence of cervical disc herniation. Unfortunately, these statistics were not statistically significant.

As it mentioned above regarding risk factors, a heavy physical workload and occupations requiring harder effort are associated with a possibility of increased occurrence of disc herniation. Longer working hours may be the factor that causes physicians to be in a higher-risk group. This operating mode may lead to a poor posture and lifestyle, and it is a risk factor for the development of cervical disc herniation.

Young-Ki et al. [76] carried out another study regarding the occurrence of cervical disc herniation and its association with age, gender, and health-insurance eligibility. However, we will only deal with the frequency distribution of this pathology between individual age groups and gender.

The number of patients increased with aging in the male and female groups. A small decrease was noticed between the 50–59 and 60–69 age groups in the female population. The frequency of cervical disc herniation was higher among female patients than male patients in every age group (Table 4) [76].

The next study was carried out by Tabesh et al. [77]. Briefly, 269 patients (102 women and 167 men) underwent a straight leg-raising test. A positive result was observed in 182 patients (67.7%). A chi-square test showed that the gender distribution between the two groups was statistically significant (*p* = 0.003). The distribution of the level of disc herniation between the two groups and the distribution of age between the two groups was also statistically significant (*p* = 0.002 and *p* <0.001, respectively). In these cases, the Fischer Exact test was used. The Mann–Whitney U-test showed that the distribution of severity between the two groups was also statistically significant (*p* < 0.001). Considering gender, the vast majority of patients were men (62.5%). The largest group regarding age distribution was the 30–39 age group (Table 5) [65].

Another study [76] showed the relationship between the occurrence of disc herniation and various factors. The number of patients with LDH in 100,000 individuals according to gender, age and health-insurance eligibility was studied and assigned to the appropriate groups. Given the results in Table 6, we suggest that the number of patients increased with age. In addition, more male patients were found in the 20–39 age group. The situation was completely different among the surveyed population over 40 years of age. Lumbar disc herniation was more common in women [76].

Another study carried out by Jong-myung et al. [78] that was based on a national database in the Republic of Korea (ROK) from January 2008 to December 2016. The number of patients with diagnosed disc herniation increased from 2008 to 2012, and then fell. The patients were divided into nine age groups; the results of this research are presented in Table 7 (percentage distribution)

Individuals were also divided according to gender. In 2008, there were higher numbers of lumbar disc herniation in female patients than in male patients in their 30s, and 1.6 times higher than patients in their 60s. In 2016, the crude incidence was also higher in female patients than male patients in the 40s group, and 1.5 times higher in the 60s group [78].

Table 8 compares the results of the studies with regard to specific age groups.

These results show that the studies carried out by Young-Ki et al. [76] and Jong-myung et al. [78] had a similar frequency distribution in terms of age groups. Tabesh [77] obtained another crude incidence of lumbar disc herniation.

Despite the fact that the above studies showed a relationship between the above-mentioned factors and disc herniation, it should be remembered that these are predisposing factors, the most important of which is their interaction with genetic factors (which play a main role in occurrence the intervertebral disc pathologies).

## 8. Prevention

As mentioned above, there are many factors that increase the risk of occurrence of some kind of intervertebral pathology. Nevertheless, we can prevent or delay their occurrence through our behavior. The status of smoking, obesity, or doing physical exercises depends only on us.

### 8.1. Avoiding Elevated BMI

Schumann et al. [79] found BMI to be associated with an increased occurrence of disc herniation. In a group of men, the most vulnerable were patients with BMI ≥ 24.30. The highest risk was observed in men who were slightly overweight (BMI of 24.3 to less than 29.21); they had more than twice the risk of disc herniation than male patients with a BMI of less than 21.88 [79].

A similar situation occurred among women, but the highest risk of disc herniation was associated with a BMI of at least 29.21. This group had more than twice the risk of a herniated disc than women whose BMI was less than 21.88. So, we concluded that maintaining a low body weight, and thus a low BMI, is one of the ways of preventing the occurrence of disc herniations. However, it is worth remembering that low BMI also leads to many other disorders [79].

### 8.2. Avoiding Smoking

As mentioned above, smoking is an independent risk factor that accelerates disc degeneration and promotes the development of lumbar disc herniation. Mattila et al. [80] reported on a prospective, 11-year follow-up of teenagers in an attempt to identify risk factors for lumbar discectomy. The results showed that daily smoking was the strongest risk factor for lumbar discectomy (with regard to male subjects) [80]. Non-smoking reduced the risk of excessive capillary contraction, and thus allowed the maintenance of proper diffusion of nutrition into the disc, which prevented the occurrence of disc herniation [60,80]

### 8.3. Stretch Often When Sitting for Long Periods of Time

Sitting for long periods of time increases the risk of occurrence of disc herniation. Sitting causes increased intradiscal pressure in the nucleus pulposus of approximately 40% compared to standing. Prolonged loading connected with sitting decreases disc hydration, and nucleus pulposus get weaker which result in disc herniation. Moreover, rapid fluctuations of load (such as between flexion and extension) have not been found to affect nucleus pulposus hydration [81]. Wilk et al. [82] observed that constantly changing position is important in promoting the flow of fluid (nutrition) to the disc. We can also decrease the risk of the occurrence of this pathology by maintaining proper posture during sitting. Callaghan & McGill [83] confirmed the statement about changing position during prolonged sitting. The passive tissues of the lumbar spinal region can relieve the load associated with sitting. Regularly getting up causes a favorable change in position, and leads to a positive clinical outcome [81,83].

### 8.4. Regular Physical Exercises

One of the ways to prevent the occurrence of intervertebral disc pathology is to do lumbar spine stabilization exercises, which improve spinal stability and athletic performance. Moreover, doing these exercises has a positive effect on musculature and activation of deep muscles and their flexibility, and on any strength deficits of the superficial muscles of the spine. These changes allow proper posture of the body to be maintained, thus reducing the risk of spinal pathologies. Lumbo-sacral stability improvement leads to reduction of compressive overloads. When examining the relationship of instability and disc degeneration, the positive effects of regular physical exercises, such as lumbar spine stabilization exercises, is a preventive factor against the occurrence of disc herniation [84].

## 9. Summary

At the turn of the 21st century, many studies were performed regarding the origin of abnormalities connected with intervertebral discs. The classical theory of aging and wear-and tear developed into one of a sophisticated multiple-causative disease, based on molecular and genetic alterations. Polymorphisms or mutations of genes appear to be associated with disc herniations, and this correlation occurs among both humans and animals. In this review, non-genetic factors were included in predisposing factors. This review also showed the importance of interaction between genetic and non-genetic factors in the occurrence and acceleration of intervertebral disc pathologies, based on the example of disc herniations. There are also some ways to prevent or delay the presence of such a pathology, eliminating the non-genetic risk factors, which usually accelerate the process of degeneration. Significant progress in molecular biology may affect the creation of genetic therapy.

## Figures and Tables

**Table 1:** Comparison of risk for cervical HIVD between HCPs (physicians and nonphysicians) and non-HCP references (general population).

Cohort (*n*)	Number (%)
Physicians (26,038)	385 (1.48%)
Non-HCPs References (26,038)	285 (1.09%)
Non-physician HCPs (33,057)	417 (1.26%)
Non-HCPs references (33,057)	303 (0.92%)

**Table 2:** Comparison of risk for cervical disc herniation between physicians and non-physician HCPs.

Cohort (*n*)	Number (%)
Physicians (26,038)	385 (1.48%)
Non-physicians HCPs (33,057)	417 (1.26%)
**Male**	
Physicians (22,181)	339 (1.53%)
Non-physicians HCPs (12,156)	146 (1.2%)
**Female**	
Physicians (3857)	46 (1.19%)
Non-physicians HCPs (20,901)	261 (1.3%)

**Table 3:** Comparison of occurrence of cervical disc herniation among physician specialists.

Specialty (*n*)	Number (%)
Internal medicine (6539)	101 (1.54%)
Pediatrics (2925)	36 (1.23%)
Obstetrics and gynecology (2158)	29 (1.34%)
Emergency medicine (534)	7 (1.31%)
Orthopedics (376)	9 (2.39%)
Surgery (3309)	47 (1.42%)
Other (10,097)	156 (1.53%)

**Table 4:** Number of patients with cervical disc herniation according to gender and age.

Age Group	Male	Female
20–29	2%	3%
30–39	5%	5%
40–49	9%	12%
50–59	13%	19%
60–69	15%	17%

**Table 5:** Frequency distribution of gender and age group of patients with lumbar disc herniation.

Gender	
Male	167 (62%)
Female	102 (38%)
Age Group	
10–19	5 (2%)
20–29	46 (17%)
30–39	92 (34%)
40–49	58 (22%)
50–59	34 (13%)
60–69	20 (7%)
70 and +	14 (5%)

**Table 6:** Frequency distribution of patients with lumbar disc herniation according to gender and age.

Age Group	Male	Female
20–29	5%	4%
30–39	7%	6%
40–49	8%	10%
50–59	10%	17%
60–69	13%	20%

**Table 7:** Frequency distribution of patients with lumbar disc herniation according to age group in 2008, 2012, and 2016

Age Group	2008	2012	2016
0–9	0.01	0.01	0.01
10–19	1.37	1.34	1.07
20–29	7.18	6.34	5.78
30–39	12.61	12.84	11.54
40–49	20.68	18.35	16.61
50–59	22.16	24.34	23.44
60–69	19.98	17.86	19.84
70–79	13.04	14.70	15.48
≥80	2.97	4.22	6.23

**Table 8:** A comparison of Tabesh et al. [77], Young-Ki et al. [76], and Jong-myung et al. [78] based on the occurrence of disc herniation in specific age groups.

Age Group	Tabesh et al. [77]	Young-Ki et al. [76]	Jong-myung et al. 2016 [78]
20–29	18%	9%	7%
30–39	37%	13%	15%
40–49	24%	18%	22%
50–59	14%	27%	30%
60–69	7%	33%	26%

## References

[1] Schnake K., Hoffmann C.-H., Kandziora F. (2012). Der zervikale Bandscheibenvorfall. Zeitschrift Für Orthopädie Und Unfallchirurgie.

[2] Amin R.M., Andrade N.S., Neuman B.J. (2017). Lumbar Disc Herniation. Curr. Rev. Musculoskelet. Med..

[3] De Cicco F.L., Camino Willhuber G.O. Nucleus Pulposus Herniation. https://www.ncbi.nlm.nih.gov/books/NBK542307/.

[4] Pfirrmann C.W.A., Metzdorf A., Zanetti M., Hodler J., Boos N. (2001). Magnetic Resonance Classification of Lumbar Intervertebral Disc Degeneration. Spine.

[5] Ikuno A., Akeda K., Takebayashi S.-I., Shimaoka M., Okumura K., Sudo A. (2019). Genome-wide analysis of DNA methylation profile identifies differentially methylated loci associated with human intervertebral disc degeneration. PLoS ONE.

[6] Weiler C., Lopez-Ramos M., Mayer H.M., Korge A., Siepe C.J., Wuertz K., Weiler V., Boos N., Nerlich A.G. (2011). Histological analysis of surgical lumbar intervertebral disc tissue provides evidence for an association between disc degeneration and increased body mass index. BMC Res Notes.

[7] Koes B.W., van Tulder M.W., Thomas S. (2006). Diagnosis and treatment of low back pain. BMJ.

[8] Gonzalez-Fiol A. (2018). Disc Herniation. Consult. Obstet. Anesthesiol..

[9] Vialle L.R., Vialle E.N., Henao J.E.S., Giraldo G. (2010). Lumbar disc herniation. Rev. Bras. Ortop. Engl. Ed..

[10] Brinjikji W., Luetmer P.H., Comstock B., Bresnahan B., Chen L., Deyo R., Halabi S., Turner J., Avins A., James K. (2015). Systematic Literature Review of Imaging Features of Spinal Degeneration in Asymptomatic Populations. Am. J. Neuroradiol..

[11] Waxenbaum J.A., Reddy V., Williams C., Futterman B. Anatomy, Back, Lumbar Vertebrae. https://www.ncbi.nlm.nih.gov/books/NBK459278/.

[12] Choi K.-S., Cohn M.J., Harfe B.D. (2008). Identification of nucleus pulposus precursor cells and notochordal remnants in the mouse: Implications for disk degeneration and chordoma formation. Dev. Dyn..

[13] Pearson J.C., Lemons D., McGinnis W. (2005). Modulating Hox gene functions during animal body patterning. Nat. Rev. Genet..

[14] Smith L.J., Nerurkar N.L., Choi K.-S., Harfe B.D., Elliott D.M. (2010). Degeneration and regeneration of the intervertebral disc: Lessons from development. Dis. Model. Mech..

[15] Ashley J.W., Enomoto-Iwamoto M., Smith L.J., Mauck R.L., Chan D., Lee J., Heyworth M.F., An H., Zhang Y. (2016). Intervertebral disc development and disease-related genetic polymorphisms. Gene Funct. Dis..

[16] Battié M.C., Videman T., Kaprio J., E Gibbons L., Gill K., Manninen H., Saarela J., Peltonen L. (2009). The Twig and functional analysis of the allelic polymorphism indegeneration†. Spine J..

[17] Feng Y., Egan B., Wang J. (2016). Genetic factors in intervertebral disc degeneration. Genes Dis..

[18] Casadei K., Kiel J. Anthropometric Measurement Contraindications. https://www.ncbi.nlm.nih.gov/books/NBK537315/.

[19] Videman T., Battié M.C. (1999). The influence of occupation on lumbar degeneration. Spine.

[20] Ahsan M.K., Matin T., I Ali M., A Awwal M., Sakeb N. (2013). Relationship between physical work load and lumbar disc herniation. Mymensingh Med. J..

[21] Kelsey J.L., Hardy R.J. (1975). Driving of motor vehicles as a risk factor for acute herniated lumbar intervertebral disc. Am. J. Epidemiol..

[22] Buckwalter J.A. (1995). Aging and Degeneration of the Human Intervertebral Disc. Spine.

[23] Frymoyer J.W. (1992). Lumbar disk disease: Epidemiology. Instr. Course Lect..

[24] Zhang Y.-G., Sun Z., Liu J., Guo X. (2008). Advances in Susceptibility Genetics of Intervertebral Degenerative Disc Disease. Int. J. Biol. Sci..

[25] Freemont A.J. (2008). The cellular pathobiology of the degenerate intervertebral disc and discogenic back pain. Rheumatology.

[26] Pelle D.W., Peacock J.D., Schmidt C.L., Kampfschulte K., Scholten D.J., Russo S.S., Easton K.J., Steensma M.R. (2014). Genetic and Functional Studies of the Intervertebral Disc: A Novel Murine Intervertebral Disc Model. PLoS ONE.

[27] Hanaei S., Abdollahzade S., Khoshnevisan A., Kepler C.K., Rezaei N. (2015). Genetic aspects of intervertebral disc degeneration. Rev. Neurosci..

[28] Wang C., Wang W.-J., Yan Y.-G., Xiang Y.-X., Zhang J., Tang Z.-H., Jiang Z.-S. (2015). MicroRNAs: New players in intervertebral disc degeneration. Clin. Chim. Acta.

[29] Kepler C.K., Ponnappan R.K., Tannoury C.A., Risbud M.V., Anderson D.G. (2013). The molecular basis of intervertebral disc degeneration. Spine J..

[30] Videman T., Battié M.C., Ripatti S., Gill K., Manninen H., Kaprio J. (2006). Determinants of the progression in lumbar degeneration: A 5-year follow-up study of adult male monozygotic twins. Spine.

[31] Battié M.C., Videman T., Levälahti E., Gill K., Kaprio J. (2008). Genetic and Environmental Effects on Disc Degeneration by Phenotype and Spinal Level: A multivariate twin study. Spine.

[32] Videman T., Levälahti E., Battié M.C. (2007). The Effects of Anthropometrics, Lifting Strength, and Physical Activities in Disc Degeneration. Spine.

[33] Varlotta G.P., Brown M.D., Kelsey J.L., Golden A.L. (1991). Familial predisposition for herniation of a lumbar disc in patients who are less than twenty-one years old. J. Bone Jt. Surg. Am. Vol..

[34] Filho R.V.T., Abe G.D.M., Daher M.T. (2020). A influência genética na degeneração discal—Revisão sistemática da literatura. Rev. Bras. Ortop..

[35] Saftić R., Grgić M., Ebling B., Splavski B. (2006). Case-control Study of Risk Factors for Lumbar Intervertebral Disc Herniation in Croatian Island Populations. Croat. Med. J..

[36] Fernandes I., Hampson G., Cahours X., Morin P., Coureau C., Couette S., Prié D., Biber J., Murer H., Friedlander G. (1997). Abnormal sulfate metabolism in vitamin D-deficient rats. J. Clin. Investig..

[37] Chan D., Song Y.-Q., Sham P., Cheung K.M.C. (2006). Genetics of disc degeneration. Eur. Spine J..

[38] Videman T., Gibbons L.E., Battié M.C., Maravilla K., Vanninen E., Leppävuori J., Kaprio J., Peltonen L. (2001). The Relative Roles of Intragenic Polymorphisms of the Vitamin D Receptor Gene in Lumbar Spine Degeneration and Bone Density. Spine.

[39] Arai H., Miyamoto K.-I., Taketani Y., Yamamoto H., Iemori Y., Morita K., Tonai T., Nishisho T., Mori S., Takeda E. (1997). A Vitamin D Receptor Gene Polymorphism in the Translation Initiation Codon: Effect on Protein Activity and Relation to Bone Mineral Density in Japanese Women. J. Bone Miner. Res..

[40] Pluijm S.M.F., Van Essen H.W., Bravenboer N., Uitterlinden A.G., Smit J.H., Pols H.A.P., Lips P. (2004). Collagen type I 1 Sp1 polymorphism, osteoporosis, and intervertebral disc degeneration in older men and women. Ann. Rheum. Dis..

[41] Annunen S. (1999). An Allele of COL9A2 Associated with Intervertebral Disc Disease. Science.

[42] Paassilta P., Lohiniva J., Göring H.H.H., Perälä M., Räinä S.S., Karppinen J., Hakala M., Palm T., Kröger H., Kaitila I. (2001). Identification of a Novel Common Genetic Risk Factor for Lumbar Disk Disease. JAMA.

[43] Mio F., Chiba K., Hirose Y., Kawaguchi Y., Mikami Y., Oya T., Mori M., Kamata M., Matsumoto M., Ozaki K. (2007). A Functional Polymorphism in COL11A1, Which Encodes the α1 Chain of Type XI Collagen, Is Associated with Susceptibility to Lumbar Disc Herniation. Am. J. Hum. Genet..

[44] Solovieva S., Lohiniva J., Leino-Arjas P., Raininko R., Luoma K., Ala-Kokko L., Riihimäki H. (2005). Intervertebral disc degeneration in relation to the COL9A3 and the IL-1ß gene polymorphisms. Eur. Spine J..

[45] Kawaguchi Y., Osada R., Kanamori M., Ishihara H., Ohmori K., Matsui H., Kimura T. (1999). Association Between an Aggrecan Gene Polymorphism and Lumbar Disc Degeneration. Spine.

[46] Takahashi M., Haro H., Wakabayashi Y., Kawa-Uchi T., Komori H., Shinomiya K., Hernigou P., Bahrami T. (2001). The association of degeneration of the intervertebral disc with 5a/6a polymorphism in the promoter of the human matrix metalloproteinase-3 gene. J. Bone Jt. Surg. Br. Vol..

[47] Dominici R., Cattaneo M., Malferrari G., Archi D., Mariani C., Grimaldi L.M.E., Biunno I. (2002). Cloning and functional analysis of the allelic polymorphism in the transcription regulatory region of interleukin-1α. Immunogenetics.

[48] Wang Z., Qu Z., Fu C., Xu F., Chen Y., Wang Z., Liu Y. (2016). Interleukin 1 Polymorphisms Contribute to Intervertebral Disc Degeneration Risk: A Meta-Analysis. PLoS ONE.

[49] Noponen-Hietala N., Virtanen I., Karttunen R., Schwenke S., Jakkula E., Li H., Merikivi R., Barral S., Ott J., Karppinen J. (2005). Genetic variations in IL6 associate with intervertebral disc disease characterized by sciatica. Pain.

[50] Wuertz-Kozak K., Haglund L. (2013). Inflammatory Mediators in Intervertebral Disk Degeneration and Discogenic Pain. Glob. Spine J..

[51] Eskola P.J., Kjaer P., Daavittila I.M., Solovieva S., Okuloff A., Sorensen J.S., Wedderkopp N., Ala-Kokko L., Männikkö M., Karppinen J. (2010). Genetic risk factors of disc degeneration among 12-14-year-old Danish children: A population study. Int. J. Mol. Epidemiol. Genet..

[52] Seki S., Kawaguchi Y., Chiba K., Mikami Y., Kizawa H., Oya T., Mio F., Mori M., Miyamoto Y., Masuda I. (2005). A functional SNP in CILP, encoding cartilage intermediate layer protein, is associated with susceptibility to lumbar disc disease. Nat. Genet..

[53] Moore R.J., Vernon-Roberts B., Fraser R.D., Osti O.L., Schembri M.A. (1996). The Origin and Fate of Herniated Lumbar Intervertebral Disc Tissue. Spine.

[54] Dong D.M., Yao M., Liu B., Sun C.Y., Jiang Y.Q., Wang K. (2007). Association between the -1306C/T polymorphism of matrix metalloproteinase-2 gene and lumbar disc disease in Chinese young adults. Eur. Spine J..

[55] Sun Z.-M., Miao L., Zhang Y.-G., Ming L. (2009). Association between the -1562 C/T Polymorphism of Matrix Metalloproteinase-9 Gene and Lumbar Disc Disease in the Young Adult Population in North China. Connect. Tissue Res..

[56] Valdes A.M., Hassett G., Hart D.J., Spector T.D. (2005). Radiographic progression of lumbar spine disc degeneration is influenced by variation at inflammatory genes: A candidate SNP association study in the Chingford cohort. Spine.

[57] Urano T., Narusawa K., Shiraki M., Usui T., Sasaki N., Hosoi T., Ouchi Y., Nakamura T., Inoue S. (2008). Association of a Single Nucleotide Polymorphism in the Insulin-Like Growth Factor-1 Receptor Gene With Spinal Disc Degeneration in Postmenopausal Japanese Women. Spine.

[58] Dor Y., Cedar H. (2018). Principles of DNA methylation and their implications for biology and medicine. Lancet.

[59] Andersen S.B., Smith E.C., Støttrup C., Carreon L.Y., Andersen M.Ø. (2017). Smoking Is an Independent Risk Factor of Reoperation Due to Recurrent Lumbar Disc Herniation. Glob. Spine J..

[60] Akmal M., Kesani A., Anand B., Singh A., Wiseman M., Goodship A. (2004). Effect of Nicotine on Spinal Disc Cells: A Cellular Mechanism for Disc Degeneration. Spine.

[61] Holm S., Nachemson A. (1988). Nutrition of the Intervertebral Disc: Acute Effects of Cigarette Smoking: An Experimental Animal Study. Upsala J. Med. Sci..

[62] Seidler A., Bolm-Audorff U., Siol T., Henkel N., Fuchs C., Schug H., Leheta F., Marquardt G., Schmitt E., Ulrich P.T. (2003). Occupational risk factors for symptomatic lumbar disc herniation; a case-control study. Occup. Environ. Med..

[63] Zhang Y.-G., Sun Z., Zhang Z., Liu J., Guo X. (2009). Risk factors for lumbar intervertebral disc herniation in Chinese population: A case-control study. Spine.

[64] Sun Z.M., Ling M., Chang Y.H., Liu Z.Z., Xu H.H., Gong L.Q., Liu J., Zhang Y.G. (2010). Case-control study of the risk factors of lumbar intervertebral disc herniation in 5 northern provinces of China. Nan Fang Yi Ke Da Xue Xue Bao.

[65] Paul C.P.L., de Graaf M., Bisschop A., Holewijn R.M., Van De Ven P.M., Van Royen B.J., Mullender M.G., Smit T.H., Helder M.N. (2017). Static axial overloading primes lumbar caprine intervertebral discs for posterior herniation. PLoS ONE.

[66] Wu Q. (2017). Intervertebral Disc Aging, Degeneration, and Associated Potential Molecular Mechanisms. J. Head Neck Spine Surg..

[67] Wood A.R., Esko T., Yang J., Vedantam S., Pers T.H., Gustafsson S., Chu A.Y., Estrada K., Luan J., The Electronic Medical Records and Genomics (eMERGE) Consortium (2014). Defining the role of common variation in the genomic and biological architecture of adult human height. Nat. Genet..

[68] Bozzoli C., Deaton A., Quintana-Domeque C. (2009). Adult Height and Childhood Disease. Demography.

[69] Heliövaara M. (1987). Body Height, Obesity, and Risk of Herniated Lumbar Intervertebral Disc. Spine.

[70] Andreasen C.H., Stender-Petersen K.L., Mogensen M.S., Torekov S.S., Wegner L., Andersen G., Nielsen A.L., Albrechtsen A., Borch-Johnsen K., Rasmussen S.S. (2007). Low Physical Activity Accentuates the Effect of the FTO rs9939609 Polymorphism on Body Fat Accumulation. Diabetes.

[71] Young A.I., Wauthier F., Donnelly P. (2016). Multiple novel gene-by-environment interactions modify the effect of FTO variants on body mass index. Nat. Commun..

[72] Knutsson B., Sandén B., Sjoden G., Järvholm B., Michaelsson K. (2015). Body Mass Index and Risk for Clinical Lumbar Spinal Stenosis: A Cohort Study. Spine.

[73] Onyemaechi N.O., E Anyanwu G., Obikili E.N., Onwuasoigwe O., E Nwankwo O. (2016). Impact of overweight and obesity on the musculoskeletal system using lumbosacral angles. Patient Prefer. Adherence.

[74] Rodriguez-Martinez N.G., Perez-Orribo L., Kalb S., Reyes P.M., Newcomb A.G.U.S., Hughes J., Theodore N., Crawford N.R. (2016). The role of obesity in the biomechanics and radiological changes of the spine: An in vitro study. J. Neurosurg. Spine.

[75] Liu C., Huang C.-C., Hsu C.-C., Lin H.-J., Guo H.-R., Su S.-B., Wang J.-J., Weng S.-F. (2016). Higher risk for cervical herniated intervertebral disc in physicians: A retrospective nationwide population-based cohort study with claims analysis. Medicine.

[76] Kim Y., Kang D.M., Lee I., Kim S.Y. (2018). Differences in the Incidence of Symptomatic Cervical and Lumbar Disc Herniation According to Age, Sex and National Health Insurance Eligibility: A Pilot Study on the Disease’s Association with Work. Int. J. Environ. Res. Public Health.

[77] Tabesh H., Tabesh A., Fakharian E., Fazel M., Abrishamkar S. (2015). The effect of age on result of straight leg raising test in patients suffering lumbar disc herniation and sciatica. J. Res. Med Sci..

[78] Jung J.-M., Lee S.U., Hyun S.-J., Kim K.-J., Jahng T.-A., Oh C.W., Kim H.-J. (2020). Trends in Incidence and Treatment of Herniated Lumbar Disc in Republic of Korea: A Nationwide Database Study. J. Korean Neurosurg. Soc..

[79] Schumann B., Bolm-Audorff U., Bergmann A., Ellegast R., Elsner G., Grifka J., Haerting J., Jäger M., Michaelis M., Seidler A. (2010). Lifestyle factors and lumbar disc disease: Results of a German multi-center case-control study (EPILIFT). Arthritis Res..

[80] Mattila V.M., Saarni L., Parkkari J., Koivusilta L., Rimpelä A. (2008). Early risk factors for lumbar discectomy: An 11-year follow-up of 57,408 adolescents. Eur. Spine J..

[81] Howell E.R. (2012). Conservative management of a 31 year old male with left sided low back and leg pain: A case report. J. Can. Chiropr. Assoc..

[82] Wilk B.R., Fisher K.L., Rangelli D. (1995). The Incidence of Musculoskeletal Injuries in an Amateur Triathlete Racing Club. J. Orthop. Sports Phys. Ther..

[83] Callaghan J.P., McGill S.M. (2001). Low back joint loading and kinematics during standing and unsupported sitting. Ergonomics.

[84] Ye C., Ren J., Zhang J., Wang C., Liu Z., Li F., Sun T.-S. (2015). Comparison of lumbar spine stabilization exercise versus general exercise in young male patients with lumbar disc herniation after 1 year of follow-up. Int. J. Clin. Exp. Med..

